# Sorption of Heavy Metal Ions of Chromium, Manganese, Selenium, Nickel, Cobalt, Iron from Aqueous Acidic Solutions in Batch and Dynamic Conditions on Natural and Synthetic Aluminosilicate Sorbents

**DOI:** 10.3390/ma13225271

**Published:** 2020-11-21

**Authors:** Jolanta Flieger, Justyna Kawka, Wojciech Płaziński, Rafał Panek, Jarosław Madej

**Affiliations:** 1 Department of Analytical Chemistry, Medical University of Lublin, Chodźki 4A, 20-093 Lublin, Poland; justynakawka@umlub.pl; 2 Jerzy Haber Institute of Catalysis and Surface Chemistry, Polish Academy of Sciences, Niezapominajek 8, 30-239 Krakow, Poland; wojtek_plazinski@o2.pl; 3 Department of Geotechnics, Civil Engineering and Architecture Faculty, Lublin University of Technology, Nadbystrzycka 40, 20-618 Lublin, Poland; r.panek@pollub.pl (R.P.); j.madej@pollub.pl (J.M.)

**Keywords:** sorbents, sorption properties, heavy metals

## Abstract

Zeolites are materials with known sorption properties. The sorption is thought to progress mainly by ion exchange with Na^+^, K^+^, Mg^2+^, Ca^2+^ or H^+^ from the zeolite exchange sites. The aim of the study was to compare the sorption properties of natural and synthetic zeolites on the example of the removal of selected metals from aqueous acidic solutions. Uptake experiments for selected ions of chromium, manganese, selenium, nickel, cobalt, and iron were performed using the batch and kinetic column methods. The sorption of the individual metal ions in mg per 1g of sorbent was determined for each sorbent. The relative affinity sequence of the examined cations toward the various sorbent was presented. The Langmuir model was used to model the adsorption equilibrium. Vermiculite under 1 mm of diameter (SF), Na-X, and Na-A were proved to be the most suitable for the individual uptake of studied metal ions. It was observed that the behavior of selenium ions differed from the remaining ones which was interpreted that selenium undergoes adsorption in the anionic form. The fixed-bed column studies were performed using Na-A, ensuring the sorption of selenium in the presence of iron(III) ions. The experiments were conducted using Na-X zeolite pre-loaded by Fe(III) as well as unmodified sorbent eluted by an equimolar mixture containing 100 ppm of Fe and Se. Obtained results prove that selenium sorption improves if other metal ions such as iron appear in the acidic solution. That efficient selenium sorption conditions can be applied to remove selenium which was recognized as toxic at higher levels.

## 1. Introduction

Heavy metals are very dangerous pollutants of the environment, mainly because they are not biodegradable and accumulate in the food chain contributing to the development of serious diseases. One of the main environmental pollutions is water pollution caused by human activity in the household, agriculture, and industry. Currently, the removal of heavy metals from an anthropogenic environment has become a priority. Many methods useful for this purpose have been described, such as chemical precipitation, ion exchange, filtration, membrane separation, reverse osmosis, phytoextraction, ultrafiltration, and electrodialysis [1,2,3,4,5]. However, there is still a need to look for more economical, safe alternative methods for removing heavy metals from the aquatic environment. In practice, adsorption techniques have found the greatest use due to the low cost and high efficiency in removing impurities of various origins such as dyes, detergents, and desiccants, whereas their acidity makes them attractive catalysts. So far, the usefulness of such sorption materials as polymer resins, natural zeolites, clays, bio-adsorbents, activated carbon, organic, and inorganic mesoporous silica has been described [6,7,8,9,10,11,12,13,14,15,16].

Clay minerals are group of hydrous layer aluminosilicates. They are commonly >2 μm, or even 4 μm in at least one dimension. Their unique properties such as high cation exchange capacities, catalytic properties, and plastic behavior when moist are results of their small size and large surface area to volume ratio [17]. Clays have been extensively applied in various applications as: colloid stabilizers, catalysts, chemical supports, coatings, drilling agents, construction materials, filling, geological applications and agriculture [18]. Additionally, there are some proofs that clays can be used as adsorbents for heavy metals and other pollutants from water and other media [19]. The removal of heavy metal ions (chromium(III), lead(II) and zinc(II) by vermiculite was studied taking into account the effect of various factors like pH value and initial ion concentration [20,21]. The performance of glauconite for purification of aqueous solutions containing heavy metal ions (lead(II), cadmium(II), zinc(II) and copper(II)), in batch and fixed-bed column adsorption systems were studied taking into account a contact time, initial concentration, and pH values [10,22]. Besides vermiculites and glauconites, bentonites are naturally occurring clay minerals which also have high adsorption capacities towards heave metals. Their performances were tested by batch experiments for removing of different heavy metals from aqueous solutions [23,24]. Great advantage of all abovementioned clay minerals is fact that they are non-toxic, easy to mine and cheap which make them eco-friendly materials.

Perlite is an inert, porous, permeable and low density volcanic material composed mainly of silica and alumina. It has highly developed surface area [25]. Due to its characteristics it can be used in various branch of applications, for example: building materials, efficient filter aid and filler in several processes and materials [26,27]. Additionally, perlites are used in heavy metals removal (for example cadmium(II), molybdenum(VI), cobalt(II) copper(II) and chromium(III)) [28].

Zeolites appear to be the most useful materials for removing metallic impurities from aqueous solutions. This is due to their physicochemical properties such as high specific surface area, porosity, and ion exchange capacity. In addition, the low cost and the possibility of recycling are not insignificant. Natural zeolites are microporous hydrated aluminosilicate minerals that have valuable interchangeable properties and most importantly are non-toxic and do not pose a threat to the environment. Their structure is based on a three-dimensional network of SiO^4-^ and AlO^4-^ tetrahedrons connected by means of oxygen atoms. The negative charge is balanced by sodium, potassium, or calcium cation. The above cations may be exchanged with other ions present in the solution [29,30]. The phenomenon of ion exchange is facilitated by internal cavities and interconnected channels of molecular size, present in the structure of zeolites [31]. Natural zeolites occur in hydrated forms, such as clinoptilolite (K_2_Na_2_Ca)_2_ [(Al_6_Si_30_)O_72_]·24H_2_O [32,33]. Their sorption abilities have already been extensively studied by many authors [34]. The main disadvantage of these materials is the fact that they have no defined chemical composition. The composition is usually variable, depending on the place of occurrence [35]. In addition, they can be mixed with quartz or clay minerals. Zeolites can be obtained by synthesis, e.g., using fly ash from coal combustion [36,37].

In order to obtain more repeatable and tunable physicochemical properties of zeolites, they are modified by introducing new functional groups. Synthetic zeolites, although more expensive, show much better activity and selectivity compared to natural ones [38,39,40,41]. Synthetic zeolites have also proved to be more advantageous in terms of heavy metal sorption. An example is NaX having exchangeable Na^+^ ions in the amount of 6.54 mmol g^−1^ [9]. Synthetic zeolites, thanks to appropriate modifications, also have the ability to absorb anions [39,40,41].

Most of the studies on heavy metal adsorption on natural and synthetic zeolites described in the literature were performed in batch conditions. The main purpose of this work is to investigate the sorption properties of natural and synthetic aluminosilicate sorbents for removing heavy metals from water. The choice of sorbents is dictated by the study of the differences in sorption performance between natural and synthetic aluminosilicates. In addition, the eco-friendliness aspect was taken into account (use of waste like fly ash after coal combustion for sorbents production, e.g., zeolites), which perfectly fits into the circular economy, which is an important element of the implementation of the principle of sustainable development. Sorption efficiency was measured under static and dynamic conditions. The competitive adsorption properties of metals on sorbents were also evaluated. The equilibrium and kinetic adsorption data were modeled with Langmuir and intraparticle pore-diffusion models, respectively.

## 2. Materials and Methods

### 2.1. Chemicals and Standards

Atomic spectroscopy standard of selenium Se (1001 ± 6 µg/mL in 4% HNO_3_) were purchased from SCP SCIENCE (MS Spektrum, Warszawa, Poland). Standard solutions of Fe (1.0 ± 0.002 g/L in dil. HCl), Mn, (1.0 ± 0.002 g/L in H_2_O), Cr (1.0 ± 0.002 g/L in dil. HCl), Ni (1.0 ± 0.002 g/L in H_2_O), Co (1.0 g/L in H_2_O) were purchased from Tritisol Merck (Darmstadt, Germany). Solutions of varying concentrations were prepared by dilution of the appropriate standard. Water purified by ULTRAPURE Millipore Direct-Q 3UV-R (Merck, Darmstadt, Germany) of the resistivity 18.2 MΩ cm was used to prepare all the aqueous solutions. To avoid glass volumetric flasks contamination of the sample the containers were leached with 65% suprapur nitric acid HNO_3_ (Merck, Darmstadt, Germany) and deionized water prior to use. The initial pH value was between 1.65–2.37 for acidic solutions (Cr, Fe, Se), and 5.66–6.89 for aqueous solutions (Ni, Mn, Co) of one-component samples with an initial concentration of 100 mg L^−1^. For multi-component sorption experiments, the initial pH value of mixture was 1.66. The pH values were monitored with CPC-105 Elmetron pH-meter.

### 2.2. Sorbents Descriptions

Vermiculite sorbents came from Kovdor (Murmansk Region, Russia). Then they were expanded and three fractions were chosen for the research—super fine (SF—to 1mm), fine (F—1–2 mm) and medium (M—2–4 mm). Glauconite was magnetically separated from tertiary sandy sediments of the Lublin Upland (Poland)—Nowodwór Mine (63–125 µm) [42]. Bentonite was a commercial product purchased form Sigma-Aldrich (St. Louis, MO, USA) and had particles diameter <0.2 mm. Perlite (<2 mm) came from western Turkey and was purchased from GEMINA (Istanbul, Turkey). Natural clinoptilolite came from Sokyrnytsya deposit (Transcarpathian region, Ukraine) [43] and has particles diameter <0.2 mm. The Na-X and Na-A (both <0.2 mm) are the synthetic zeolites which were produced in the hydrothermal conversion of fly ash after coal combustion [44,45].

### 2.3. Batch Studies

For the batch experiments, an appropriate quantity of stock solution (100 ppm) was adjusted to 12 mL with distilled water mixed with 0.3 g of sorbent that was kept at different temperatures for various time. The contact time was set after the preliminary kinetics tests that showed that equilibrium was reached within 30 min at room temperature (23 ± 0.2 °C). The mixture was shaken thoroughly for 60 min by Mini-Rotator BIO RS-24 (Biosan, Poland) with maximum rotation movement (30 RPM). The metal content by atomic absorption spectrometry was determined in aliquots of the upper phases after equilibration the samples and filtration to remove insoluble material. To select appropriate filter, the samples (3 ppm solution) were filtered through a KX Syringe Filter Nylon membrane filter (13 mm, 0.45 µm) Whatman (Maidstone, UK) and Whatman No. 42 filter paper. Adsorption of metals on the filters which may produce appreciable error has been checked before the analysis of the samples. It appeared that Nylon membrane filter adsorbed less than 5% of the most metals from acidic solutions. That is why, further filtration experiments have been performed by the use of the Nylon membrane filter. The equilibrium (*c_eq_*) concentions were determined with the atomic absorption spectrometer (AAS) (Analytik Jena, Jena, Germany). The amount of metal adsorbed (*q*) onto the sorbents was calculated by the use of the following formula:

(1) $$q=\left(c_{0}-c_{eq}\right)\frac{V}{m}$$

where: *q* is the amount of adsorbed in (mg·g^−1^), *c*_0_ is the initial concentrations of metal ion in the solution (mg·L^−1^), m is the sorbent mass (g) and V is the solution volume (L).

The removal efficiency of metal ions from the solution (*R*) was calculated by the use of the following formula:

(2) $$R=\frac{c_{0}-c_{eq}}{c_{0}}\cdot 100\%$$

### 2.4. Dynamic Operation Condition

For fixed bed column studies, an empty SPE polypropylene tube with a 65 mm length and a diameter of 12 mm was packed with 0.3 g of zeolite Na-X. Experiments utilizing Na-X zeolite pre-loaded by Fe(III) were conducted by transferring 12 mL of 100 ppm solution of Fe to the tube using a volumetric pipette and then a vacuum was applied to the column outlet. After that 100 ppm solution of Se was loaded onto the column. 5 mL portions of effluent were collected and separately analyzed by atomic method absorption spectrometry (AAS). Experiments utilizing unmodified Na-X zeolite were conducted by transferring an equimolar mixture containing 100 ppm of either Fe or Se to the tube. The eluted portions of samples each at the volume of 5 mL were tested by AAS. The efficiency of the sorbent was calculated in terms of *c*/*c*_0_ (*c* = effluent metal ions concentration and *c*_0_ = influent metal ions concentration) as a function of the volume of the eluate for a given bed mass (breakthrough curve).

### 2.5. XRF, XRD and SEM

Chemical composition of sorbents was determined using Energy Dispersive X-ray Fluorescence Spectroscopy by means of an Epsilon 3x ED-XRF spectrometer (Panalytical, Malvern, UK) with an X-ray tube equipped with Rh anode with a maximum power of 50 kV as the excitation source. All the samples were subjected to standard loss on ignition test (LOI). The results were taken into account during calculation with respect to the LOI values. Powder XRD method was used to determine the phase composition of sorbents using a Panalytical X’pert MPD diffractometer (with a PW 3050/60 goniometer) (Malvern, UK), a Cu lamp and a graphite monochromator. The identification of mineral phases was based on the PDF-2 release of the 2010 database, formalized by the ICDD. The chemical composition (EDS) in microarea and microphotographs (SEM) of materials were investigated using a Quanta 250 FEG Scanning Electron Microscope by FEI (Almelo, The Netherlands).

### 2.6. Surface Area Measurements

The specific surface area was calculated with BET method using Automatic Sorption Analyzer Porosimetry system 2020 comprising the pressure transducer (Micromeritics, Norcross, GA, USA). The sorbent samples were outgassed at 250 °C for 12 h on the degas port of the analyzer. The sorption isotherms were created by adding nitrogen onto the sorbent at 77 K.

### 2.7. AAS

Contents of metals have been evaluated by the use of flame atomic absorption spectrometry. High-Resolution Continuum Source Atomic Absorption Spectrometer ContrAA700 (Analytik Jena, Jena, Germany) with a 300 W xenon short-arc lamp as a continuum radiation source was applied. The instrument was operated using Aspect CS 2.0.0 software (Analytik Jena, Jena, Germany). Acetylene was used as the fuel, while the oxidant was air. The measurements were performed at λ = 248.327 nm (Fe), 279.482 nm (Mn), 357.869 nm (Cr), 232.003 nm (Ni), 196.027nm (Se), 240.725 nm (Co). The measurements have been performed eleven times for each sample. Quantification was based on the calibration curve (r = 0.9999) estimated for each standard solution in the range of 0.03–3.00 mg L^−1^ for Fe, 0.105–1.50 mg L^−1^ for Mn, 1.19–7.00 mg L^−1^ for Cr, 0.18–6.00 mg L^−1^ for Ni, 0.3–30.0 mg L^−1^ for Se, 0.8–4.0 mg L^−1^ for Co. The effluent selenium concentration in the fixed bed column studies was measured by the use of high resolution continuum source (HR CS) Graphite Furnace Atomic Absorption (GFAA, (Analytik Jena, Jena, Germany). The detection limits (DLs) calculated using 3-Sigma (3σ) method were the following: 7.0 µg L^−1^ (Fe), 1.0 µg L^−1^ (Mn), 5.0 µg L^−1^ (Cr), 5.8 µg L^−1^ (Ni), 500 µg L^−1^ (Se), 5.0 µg L^−1^ (Co).

### 2.8. Adsorption Isotherm Model

The intraparticle pore-diffusion model was accepted to describe the non-equilibrium process of sorbate adsorption onto the porous surface materials. The following mass-balance equation using the radial coordinate r can be applied [46].

(3) $$\frac{\partial c}{\partial t}=\frac{D}{\tau }\left(\frac{\partial^{2}c}{\partial r^{2}}+\frac{2}{r}\frac{\partial c}{\partial r}\right)-\frac{\rho }{\varepsilon_{P}}\frac{\partial q}{\partial t}$$

where *t* is the time, *ρ* is the sorbent particle density, *ε_P_* is the particle porosity, *D* is the diffusion coefficient of sorbate in the solution and *τ* is the dimensionless tortuosity factor. The local concentrations of the sorbate within a particle in the solution, *c*, and in the adsorbed phase, *q* are related to their equilibrium relationship. This local equilibrium assumption can be expressed as:

(4) $$q=\phi (c)=\frac{q_{\max}K_{L}c}{1+K_{L}c}$$

where *φ*(*c*) is a general adsorption isotherm equation; the r.-h.-s. of the above equation is represented by the Langmuir model, used in our study to model the adsorption equilibrium. The *q*_max_ is the monolayer capacity and *K_L_* is the Langmuir constant. The boundary conditions following from the symmetry of the spherical particle are:

(5) $${\left(\frac{\partial c}{\partial r}\right)}_{r=0}=0\;\mathrm{and}$$

(6) $$c(r=R_{0})=c_{b}$$

where *R*_0_ is the sorbent particle radius. The initial condition for Equation (1), satisfying the experiment course (adsorption process) is:

(7) $$c(0\leq r\leq R_{0})=0\;\mathrm{at}\;t=\;0$$

The average amount adsorbed onto the particle surface, $\bar{q},$ can be found by averaging the amount adsorbed in a given point of the particle over the volume of the particle:

(8) $$\bar{q}(t)=\frac{3}{R_{0}}\int_{0}^{R_{0}}r^{2}q(r,t)dr$$

Note that many simplified mathematical forms of Equation (3) exist. However, they are not applicable to the systems under our investigation due to a series of deviations from ‘idealized’ conditions assumed in order to derive corresponding analytical relationships; in particular, the conditions about the linear equilibrium adsorption isotherm and the constant sorbate concentration in the bulk solution are not fulfilled. Therefore, we used the full form of partial differential Equation (3). All numerical solutions presented here were obtained using the in-built PDE solver package ‘NDSolve’ in Mathematica 8.1™ software Wolfram Research Inc. (Champaign, IL, USA).

## 3. Results and Discussion

### 3.1. Sorbent Characteristics

Chemical composition of studied sorbents is presented in Table 1. The dominant component of all materials is silica, which, depending on the type of sorbent, ranges from 42.52% for vermiculite M to 73.82% for perlite. Vermiculites and bentonite are characterized by a high content of MgO while in other cases, a significant amount of Al_2_O_3_ is observed, even 31.59% for zeolite Na-A. In case of glauconite, there is high content of Fe_2_O_3_—25.01%. An increased content of this component was also found in all vermiculite materials tested (9.61–10.55%).

**Figure 1:**
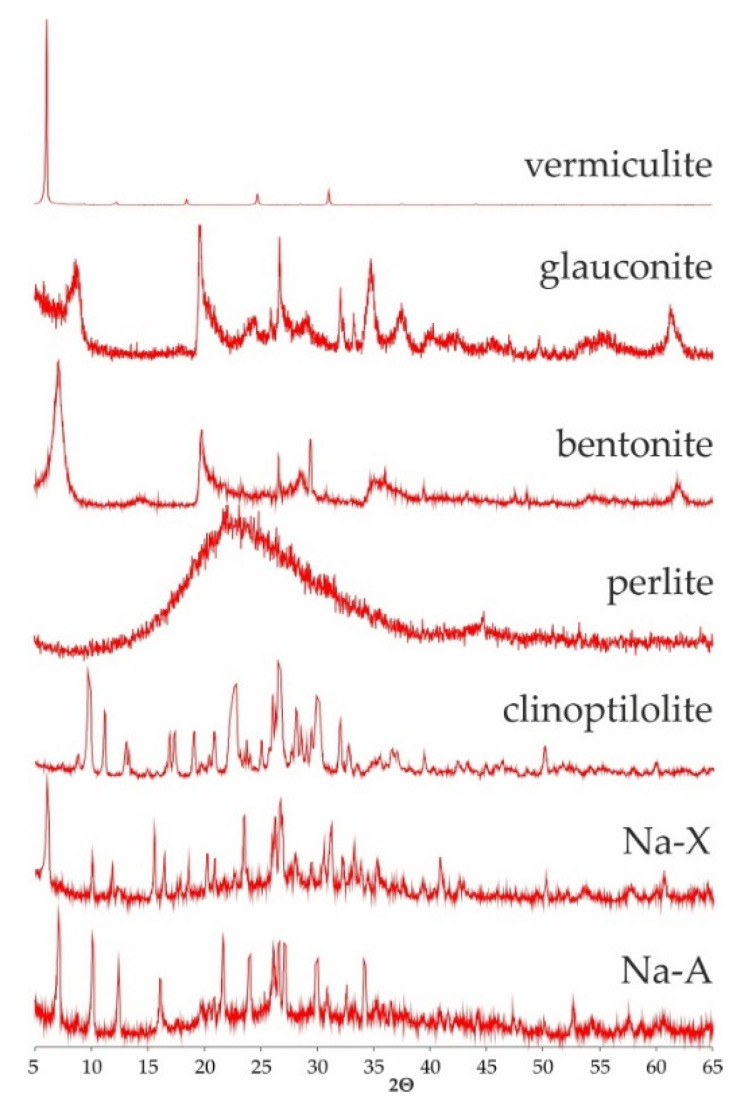
Diffractogramms of studied sorbents.

Mineral phase of the studied srobents was showed on Figure 1. All sorbents (besides perlite) were identified after their characterictics diffraction reflexes d_hkl_: vermiculites (14.28, 4.57, 4.46 Å—regardless of the fraction), glauconite (10.10, 4.53 and 3.33 Å), montmorillonite as a main component of bentonite (12.82, 4.51, 3.24), clinoptilolite (9.01, 7.94, 5.13 Å), Na-X (14.41, 8.81, 7.53 Å) and Na-A (12.20, 8.86, 7.08 Å). Perlite, as an amorphous substance, does not show characteristic interplanar distances (d_hkl_). Its diffractogram shows a clearly elevated background level between 15 and 35° 2 theta angles.

Specific surface areas for studied sorbents were as follows: 14 m^2^/g (vermiculite), 47 m^2^/g (glauconite) 36 m^2^/g (bentonite), 13 m^2^/g (perlite), 17 m^2^/g (clinoptilolite), 158 m^2^/g (Na-X), 70 m^2^/g (Na-A).

### 3.2. Sorption Properties at Batch Conditions

**Figure 2:**
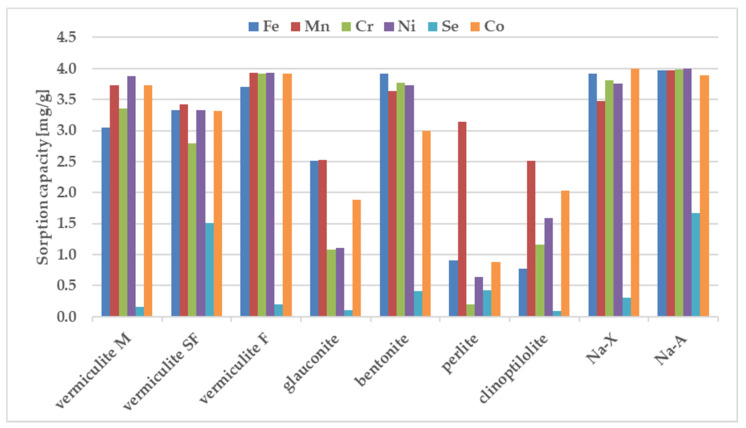
The sorption capacity of the investigated sorbents for metal ions, estimated in batch study. Initial ion concentration *c*_0_ = 100 mg L^−1^, volume of solution = 12 mL, mass of zeolite = 0.3 g, contact time = 1 h. The measurements were conducted at the room temperature (23 ± 0.2 °C).

**Figure 3:**
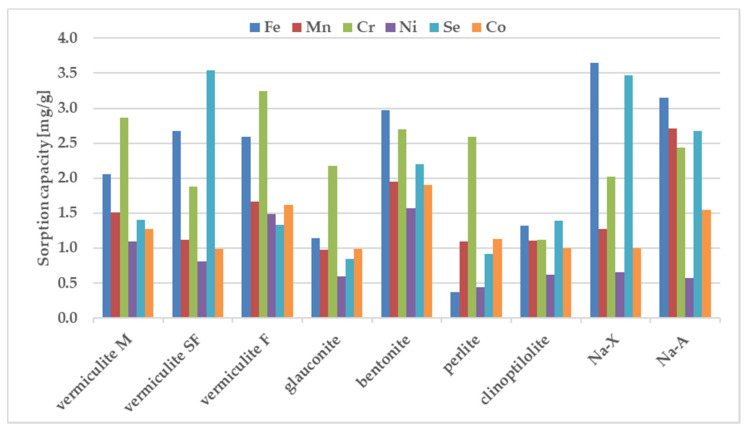
The sorption capacity of the investigated sorbents towards mixture of ions, estimated in batch study. Initial ion concentration c_0_ = 100 mg L^−1^, volume of solution = 12 mL, mass of sorbent = 0.3 g, contact time = 1 h. The measurements were conducted at the room temperature (23 ± 0.2 °C).

The sorption behavior of the examined zeolites for metal ions were determined under static conditions. The batch method involved contacting the solid phase with aqueous solutions at concentration of 100 ppm. A comparative graphs representing the amount of ions adsorbed per unit mass of adsorbent and the removal efficiencies are presented in Figure 2 and Figure 3.

The results in Figure 2 and Figure 3 show that the removal efficiency varied for each sorbent but for almost all ions is the highest for sorbent Na-X, Na-A and vermiculite SF. According to the equilibrium studies, the selectivity sequence is very similar for above three materials. The sorption ability of Cr^3+^, Mn^2+^, Ni^2+^, Co^2+^, Fe^3+^ is almost maximum, whereas percentage removal of selenium in all three sorbents is very poor. It is known that metal cations exist in aqueous solutions in the form of high-spin water complexes. Ionic radii of hydrated ions for Cr^3+^, Mn^2+^, Ni^2+^, Co^2+^, Fe^3+^ ions are of similar magnitude and equal to: 0.2192 ± 0.0013; 0.2106 ± 0.0022, 0.2061 ± 0.0014; 0.1969 ± 0.0032, 0.2031 ± 0.0019 nm respectively [47]. The above radii are comparable with the free dimensions of the channels present in zeolites, thanks to which ion transport and exchange are easy. The exception is selenium, which shows the weakest sorption on all sorbents. The reason is the selenium ion speciation observed in solutions containing nitric acid. The most abundant form of selenium is an anion, namely SeO_4_^2-^ with a much larger ionic radius of 0.395 nm, which is nearly twice as large in comparison to the remaining metal ions. The opposite charge implies the hindered progress of ion-exchange-based sorption whereas the larger dimensions are an obstacle to the selenium transport through the zeolite pores.

### 3.3. Sorption in Multi-Component Systems

Ion exchange responsible for sorption is competitive. The selectivity obtained in systems with one species is completely different in comparison to the results obtained in the case when the zeolite comes into contact with a mixture of ions of equal concentrations.

The selectivity sequence of the studied metals with respect to the investigated sorbents can be expressed as:

vermiculite M: Cr > Fe > Mn > Se > Co > Ni; vermiculite SF: Se > Fe > Cr > Mn > Co > Ni; vermiculite F: Cr > Fe > Mn > Co > Ni > Se; glauconite: Cr > Fe > Mn > Co > Se > Ni; bentonite: Fe > Cr > Se > Mn > Co > Ni; perlite: Cr > Co > Mn > Se > Ni > Fe; clinoptilolite: Se > Fe > Cr > Mn > Co > Ni; Na-X: Fe > Se > Cr > Mn > Co > Ni; Na-A: Fe > Mn > Se > Cr > Co > Ni.

**Figure 4:**
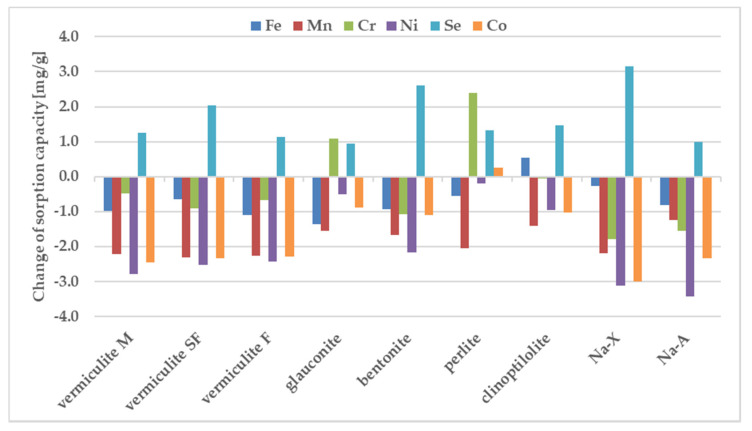
Change of sorption under influence of other ions, estimated in batch study. For experimental conditions see the label for Figure 2.

Chromium and iron displayed the highest sorption values, whereas Co and Ni were retained weaker than the other tested metals. Only vermiculite SF demonstrated the preference for strong sorption of Se. The selectivity sequence of studied metals with respect to zeolite is usually attributed to differences in metal ion properties such as: the hydrolysis constant, the atomic weight, the ionic radius, the hydration radius. The sorption preference exhibited by the investigated sorbents may be also attributed to pH value measured after mixing the components. Differences between the sorption of individual metals from samples containing individual components and from the ion mixture are shown in Figure 4.

The sorption of Co, Mn, and Ni from a mixture of all elements have been reduced to the largest degree compared to their sorption from solutions containing a single metal in the case of all zeolites. Considering sorption from a multicomponent mixture it should be pointed out that pH before mixing with zeolites is just 1.66, whereas one-component solutions of the above metals were close to neutral (5.66–6.89). In these cases, when the pH value drops dramatically from 5.6–7.0 for one-component solutions into 1.7 for all metal composite samples, the competitive participation of hydrogen ions for binding sites on the sorbent surface, making them much better candidates than metal ions for electrostatic and inner-sphere surface complexation reactions. That is why their sorption decreases at acidic solutions. Sorption of Cr and Fe does not change significantly in most zeolites except for glauconite and perlite. In the comparison presented in Figure 4, the significant sorption of Se from the multi-component mixture is noteworthy, while it was negligible from a solution containing a single metalloid. The process of metal sorption by sorbents is mainly through ion exchange. This mechanism involves the exchange of cations, mainly sodium, potassium, magnesium and calcium, due to the possibility of free migration of ions through the pores and channels of sorbent. A high concentration of metal ions can therefore cause rapid saturation of sorption sites neutralization of the negatively charge binding sites. Thus, the non-negligible sorption of selenium observed in the case of multicomponent systems can be explained in terms of non-specific binding, occuring possible with the non-direct contribution of already bound, positively charge metal ions. The high magnitude of Se sorption is observed for all tested materials, especially for vermiculite SF, bentonite and Na-X.

### 3.4. Sorption Kinetics

**Figure 5:**
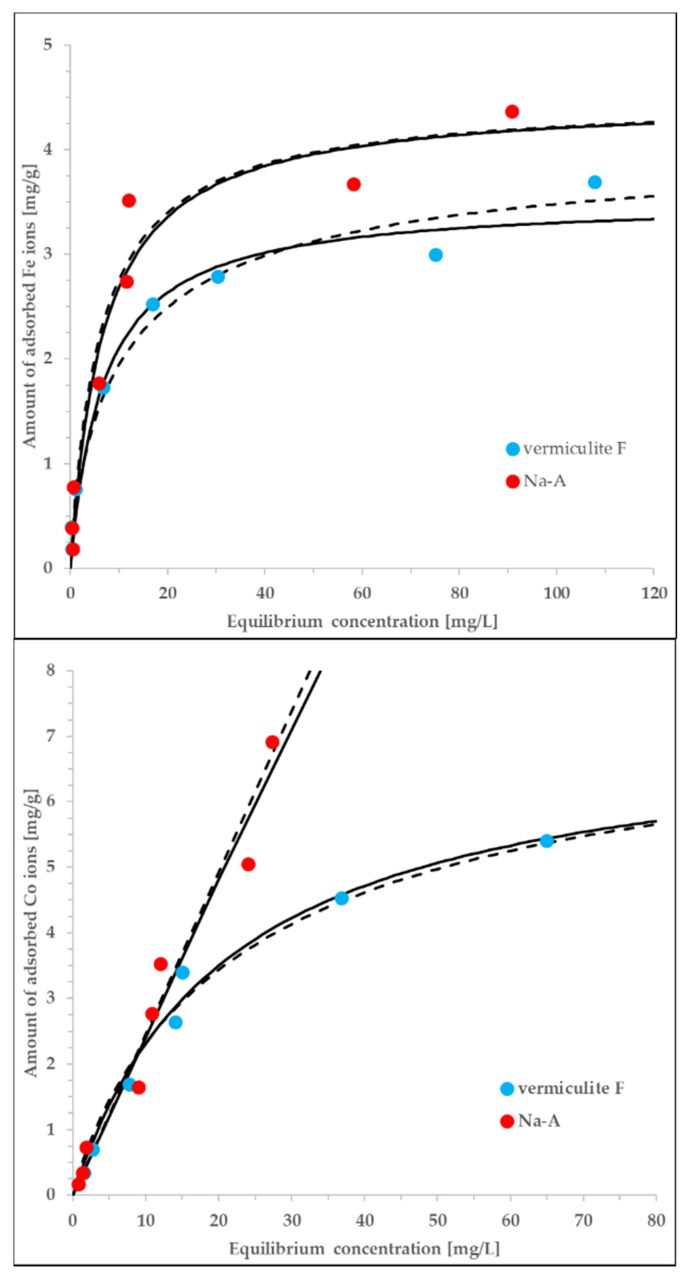
The experimental data measured for vermiculite F (C) and Na-A (J) interacting with either Co or Fe ions., equlibrium data correlated by both the Langmuir (solid lines) and Langmuir-Freundlich (dashed lines) equations. The adjusted exponents of the LF equation vary from 0.72 to 1.23, i.e., are close to unity. The parameters of the Langmuir model are discussed in the text. The rest of the details is given in the main text.

The experimentally measured equilibrium adsorption isotherms for selected systems are graphically illustrated in Figure 5. The simplest, two-parameter Langmuir model offers a satisfactory correlation of the experimental data, at the same time providing the physical interpretation of the process as the adsorption onto relatively energetically homogeneous surface. Alternative models of equilibrium adsorption (e.g., Langmuir-Freundlich, Toth) are represented by three-parameter equations, thus, the quality of the corresponding correlations may only be higher. Additionally, we have used the Langmuir-Freundlich model against the same data sets. We have checked both the fit quality and the values of adjusted parameters to conclude that the both tested models correlate the experimental data with nearly the same quality. More precisely, the determined values of the exponent in the LF equation are close to unity, which confirms the applicability of the initially chosen Langmuir model.

The adjustment of the data offered by the Langmuir model is satisfactory (the values of determination coefficient, R^2^, vary in the range of 0.955–0.989). According to the assumptions underlying this model, the adsorbent surface is energetically homogeneous with respect to the interactions with adsorbate and, at the same time, the adsorption is monolayer. The determined Langmuir constants are much higher in the case of Fe ions (0.151–0.162 L/mg) in comparison to the Co ions (0.00180–0.0440 L/mg). On the contrary, the estimated monolayer capacities are higher for the Co ions (1.35–138 mg/g) than for the Fe ions (3.52–4.42 mg/L). The exceptionally high value of q_max_ obtained for the Co/Na-A is probably the result of low concentrations at which the sorption equilibrium was measured. Nevertheless, independently of the associated inaccuracies, the highest sorption capacity is inherent to this system. The obtained differences in adjusted parameters are more ion-specific rather than sorbent-specific. Due to extremely similar ionic radii of involved metal cations [47] these differences can be explained only on a ground of unknown molecular mechanisms resulting in specific sorbate-sorbent interactions.

**Figure 6:**
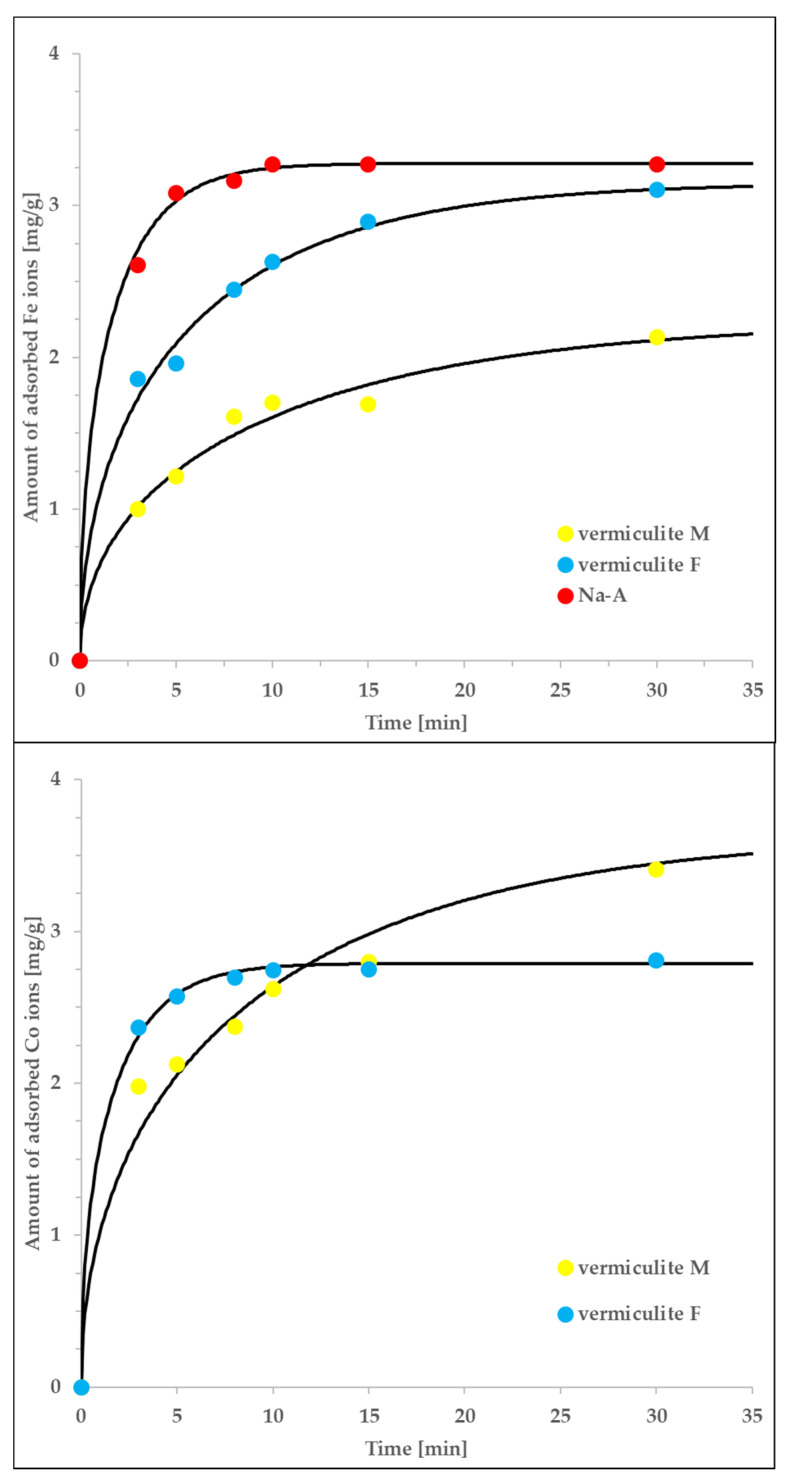
The agreement of the intraparticle pore-diffusion model (solid lines), with the non-equlibrium experimental data measured for vermiculite M, vermiculite F and Na-A interacting with either Co or Fe ions. The rest of the details is given in the main text.

The associated kinetic adsorption isotherms measured for selected systems are shown in Figure 6. The experimental data can be accurately described by the intraparticle pore-diffusion model, mathematically expressed by Equations (3)–(8). Note that, in spite of non-trivial procedure of numerically solving the associated equations, the only unknown constant is the tortuosity factor. Its value should be dependent only on the sorbet type and vary within a relatively low range of 1–100, according to assumed mechanism of diffusion (larger values would suggest significant contribution of surface diffusion whereas smaller ones are impossible for physical reasons). The values of remaining parameters were known in advance either as elements of technical procedure or from the accompanying equilibrium measurements and analysis (see the paragraph above).

The determined tortuosity factors are equal to: τ = 2.3 (for the Co/vermiculite F system), τ = 5.9 (for the Fe/vermiculite F system) and τ = 85.5 (for the Fe/Na-A system). The first two values are relatively close to each other and typical for pore diffusion in micro/mesoporous systems. Moreover, their range confirms the applicability of the intraparticle pore-diffusion model and suggests the pore-diffusion-driven kinetics of adsorption. The higher value of τ obtained for the Fe/Na-A system is the result of exceptionally small diameter of Na-A particles (in comparison to those of vermiculite F; 66 μm vs. 0.5 mm). We expect that the actual, ‘effective’ diameter was higher, due to possible aggregation of sorbent particles; this assumption would explain such high value of τ. Additionally, we expect that in zeolite microporous systems, the other types of diffusion may occur in parallel (e.g., both surface diffusion and pore diffusion). Therefore, Equation (3) may represent several different physical processes and the determined, diffusion coefficient may be a superposition of those characteristic of both surface- and pore diffusion. On the other hand, the pore diffusion process is expected to be the dominating one which is confirmed by the range of determined tortuosity factors [48].

Finally, let us mention that although the analogous numerical procedure can be performed also for vermiculite M, due to the lack of associated equilibrium parameters, we were unable to recover the tortuosity factor values (they are convoluted with several further parameters). Nevertheless, the technical quality of the fits is good, which, in combination with the knowledge about the system’s features, allows to state that the intra-particle pore-diffusion mechanism controls the adsorption kinetics also in this case.

### 3.5. Fixed Bed Column Study

The conducted experiments of sorption of metal ions from single-component solutions and from mixtures on various sorbents allow to formulate the following conclusions: (i) Cr, Fe, Co, Mn and Ni ions are sorbed from single-component solutions on most of the tested sorbents; (ii) selenium from solutions with a pH < 2 hardly sorbs at all; (iii) selenium sorption improves if other metal ions appear in the acidic solution.

**Figure 7:**
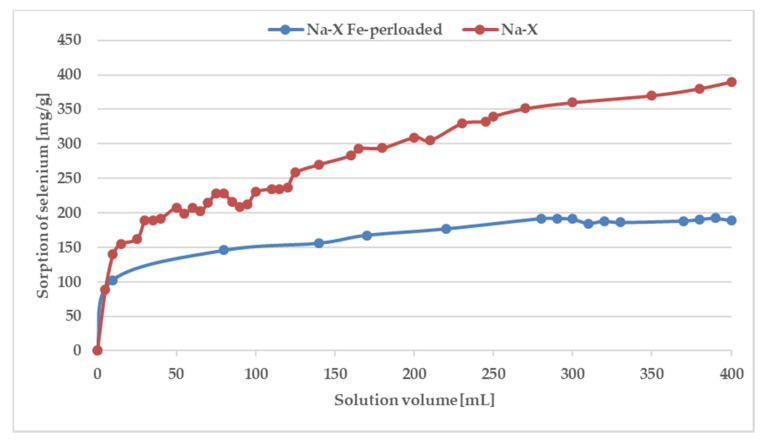
Breakthrough curves for selenium adsorption on Na-X and Na-X perloaded by iron.

Although ion exchange is widely recognized as the mechanism responsible for the sorption of metals from aqueous solutions by sorbents, it is difficult to explain selenium sorption by this mechanism. The selenate ions do not undergo ion exchange due to the negative charge of an involved ion. However, there is a possibility of electrostatic interactions with previously adsorbed ions of other metals and inner-sphere surface complexation reactions. To prove this mechanism, experiments were carried out in dynamic conditions. Furthermore, although batch studies estimate the sorption capacity of the sorbents, column operations are also important from a practical point of view. The obtained curves are presented in Figure 7.

**Figure 8:**
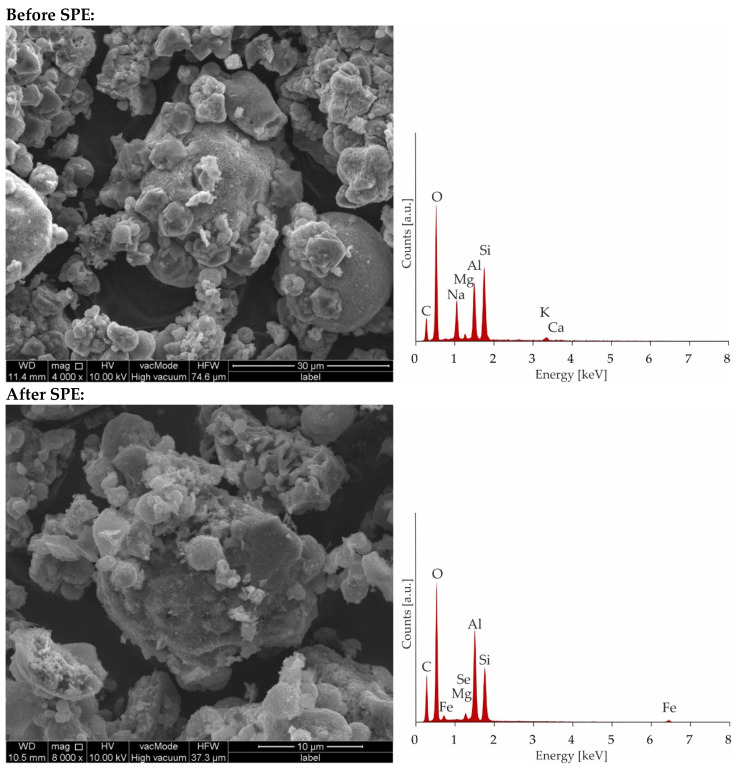
Microphotographs and EDS charts of zeolite Na-X before and after SPE.

As can be seen, the saturation has not been achieved even after the addition of 400 mL of a solution containing 100 ppm of Fe and Se. The breakthrough curve was not obtained because of the precipitation of Fe_2_(SeO_4_)_3_ characterizing by low solubility. The p_ks_ value of iron(III) selenate(VI) is as high as 23.19 [49,50] therefore in solution after mixing of solutions containing 100 ppm of ions, the product of the molar concentration of ions (1.53 × 10^−15^) does not exceed the solubility product constant, and precipitation has not been observed. However, during the SPE procedure because of local pre-saturation caused by electrostatic interactions, this process can explain the unexpected shape of the breakthrough curve. In case of preloaded sorbents by iron ions after addition of 250 mL of solution with selenate(VI) ions sorption is stabilized at almost constant level. To confirm sorption mechanism, SEM-EDS analysis was applied (Figure 8) As can be seen in Figure 8 it is difficult to notice morphological changes in the structure of investigated zeolite, however graphs illustrating the elemental content clearly show and confirm sorption of iron occurring by ion-exchange process providing to displacement of K, Ca, Mg, and Na. Unfortunately, changes in Se content are not possible to notice because of the chemical interferences from Mg and Al, present sorbent before sorption [51]. The presence of Se in Na-X after sorption was confirmed by ED-XRF analysis and Se content after SPE procedure increased from 0.0% up to 0.30%.

So far several techniques have been used to remove Se from solutions such as ion-exchange, bioremediation, adsorption, and coprecipitation. However, ion-exchange resins appear to be not efficient with an increasing concentration of competitive ions [52,53].

In the case of adsorption on the mineral surface, retention is unstable because the surrounding environment decreases already adsorbed ions [54]. Tokunaga and Takahashi proposed the incorporation of both Se(IV) and Se(VI) anions in mineral lattice during coprecipitation with toxic barite (BaSO4) [55]. Another reagent commonly uses in co-precipitation of selenium (IV) ions is ammonium pyrrolidinedithiocarbamate (APDC) [56].

The determination of trace amounts of Se utilizes high sorption capacity of solid materials such as non-polar (C-18) sorbent [57], nano-Al_2_O_3_ [58], modified activated carbon [59], cetyltrimethylammonium bromide (CTAB)-modified alkyl silica [60], Dowex 1X2 resin [61], dithiocarbamate loaded polyurethane foam [62], or dithizone sorbent [63]. Sitko et al. proposed, the preconcentration of Se using multiwalled carbon nanotubes (MWCNTs) and APDC as a chelating agent in dispersive solid-phase microextraction (DSPME) [51]. Furthermore, it was proven that synthetic and natural iron oxy-hydroxides such as magnetite, hematite, and goethite posses high sorption capacities for selenium [64]. The authors observed that the selenium sorption increases at acidic environment due to the predominance of the selenium species Se(IV) and Se(VI). The Na-X zeolite/Fe(III) system proposed in the current study can be safely applied for retention of selenium which is important considering the fact that selenium is widely recognized as an essential dietary component with numerous beneficial effects on health, but at higher levels causes toxic effects (e.g., drinking water limits of 0.05 mg/L and 0.01 mg/L in the United States and Japan, respectively) [65].

## 4. Conclusions

Different sorbents belonging to natural and synthetic aluminosilicates were used for the removal of several different metal ions such as chromium, manganese, selenium, nickel, cobalt, and iron from aqueous solutions. The obtained sorption capacities varied depending on the type of both sorbents and ions.

Among the tested sorbents, glauconite, perlite, and clinoptilolite showed the lowest sorption capacity in relation to all metal ions. The experimental kinetic and equilibrium data can be accurately described by the intraparticle pore-diffusion model and Langmuir adsorption isotherm, respectively.

The Na-X zeolite appeared to be an effective selenium sorbent. When Na-X zeolite was preloaded with Fe(III) ions, the saturation of the sorbent was achieved under dynamic conditions. However, in the case of the presence of Fe(III) ions in the solution containing selenium, the effect of sorbent saturation was not achieved due to the precipitation of Fe_2_(SeO_4_)_3_.

The proposed method of Se elimination from water samples is attractive because it uses zeolites, which is obtained from waste such as fly ash, does not require toxic reagents, and ensures efficient selenium sorption in an acidic environment, even without prior modification of the sorbent material.

## Figures and Tables

**Table 1:** Chemical composition of the adsorbents assayed by a quantitative X-ray fluorescence spectrometry (XRF).

Material	Na_2_O	MgO	Al_2_O_3_	SiO_2_	P_2_O_5_	K_2_O	CaO	TiO_2_	Fe_2_O_3_	LOI
vermiculite M	0.00	26.62	9.63	42.52	0.00	0.09	0.55	0.71	10.16	9.59
vermiculite SF	0.00	26.85	10.17	42.75	0.08	0.25	0.86	0.71	9.61	8.57
vermiculite F	0.00	27.09	10.14	44.02	0.00	1.20	0.48	0.65	10.55	5.69
glauconite	0.00	2.58	6.13	47.77	2.23	7.24	3.55	0.27	25.01	5.05
bentonite	0.00	17.96	6.65	56.17	0.00	2.12	3.11	0.45	3.71	9.67
perlite	1.92	0.00	11.05	73.82	0.00	7.47	1.56	0.22	2.32	1.55
clinoptilolite	0.00	0.53	9.54	71.67	0.02	3.77	3.59	0.21	1.85	8.69
Na-X	5.08	1.67	22.32	42.96	0.16	3.02	3.20	1.53	7.61	12.15
Na-A	2.56	0.07	31.59	48.64	0.03	1.63	2.84	0.59	2.19	9.62
